# The Treatment of Sciatica with Quinine and Urea Hydrochloride

**Published:** 1916-05

**Authors:** J. R. Garner

**Affiliations:** Atlanta, Ga.


### THE TREATMENT OF SCIATICA WITH QUININE AND UREA HYDROCHLORIDE. REPORT OF SIX CASES.

By J. R. Garner, M. D., Atlanta, Ga.

I began using this method of treatment in April, 1914, and while my experience does not include as many cases as I would like to have had on which to base a report, the results obtained in the six cases which I shall describe fully justify a continuance of the procedure.

My reasons for taking up Quinine and Urea Hydrochloride were that every other remedial agent had failed me and I wanted some other means than the use of morphine for the relief of my patients. Cocaine injected into a nerve trunk produces anesthesia more than sufficient for performing operations, but cocaine is toxic and its effect soon passes off. On the other hand, Quinine and Urea Hydrochloride is not toxic and der certain conditions its effect lasts for several days. I em-employ the sterilized solutions supplied by Parke, Davis & Co., put up in hermetically sealed glass ampoules.

Precautions are taken to have the syringe, the site of injection and the operator's hands surgically clean. The point of the needle is first thrust through the skin and a few drops of the solution expelled at that point. Then, after a few moments, the needle may be pushed into the nerve without causing great pain and the solution injected slowly. The point I usually select is where the great sciatic nerve emerges from the pelvis through the great sacro-sciatic foramen.

Case 1.—W. H. M., a switchman, 39 years old, on March 29, 1914, fell from the top of a car and sustained a severe contusion of the left hip and thigh, and general bruises. April 2nd he developed a typical case of sciatica, with intense pain along the course of the nerve. My diagnosis was traumatic neuritis of the sciatic nerve.

After trying routine measures without result I injected ten Cc. of the one-per-cent. Solution Quinine and Urea Hydrochloride directly into the sciatic nerve on the ninth of April. Three hours thereafter the patient was more comfortable and the following day was free from pain. On the 14th the pain recurred when a second injection was given. Relief was experienced until the 27th, at which time the third injection was administered. The man returned to his employment on the first of May and has had no return of the sciatica.

Case 2.—J. W., a laborer, aged 28 years, wrenched his left leg and hip several days before he applied to me for treatment, May 23rd, 1914. After satisfying myself that his was a case of sciatica I injected ten Cc. of the one-per-cent. Solution of Quinine and Urea Hydrochloride into the nerve. This relieved the pain in a few hours and it did not recur until the 29th when a second injection was necessary. June 6th the man resumed his occupation and the sciatica has not returned.

Case 3.—Mrs. L. H. W., 32 years old, has had rheumatism for several years. When I first saw her, March 3rd, 1915, she was suffering severe pain along the course of the sciatic nerve. I made a diagnosis of rheumatism with sciatica. Rheumatism Phylacogen and salicylates were used, and after ten days' treatment the patient was relieved of the rheumatic symptoms. However, the sciatica continued without abatement. March 15th, I injected Quinine and Urea Hydrochloride into the nerve. The following day the pains were much less severe, but had disappeared entirely on the 17th. On account of a recurrence on the 21st a second injection was performed, and on the 26th a third was given, which afforded complete relief. The patient felt that a pain was developing in the nerve February 5th,

1916, at which time a single injection was sufficient to relieve her completely, and she has had no recurrence since.

Case 4.—J. A. A., a salesman, 27 years of age, had had sciatica for a long time, possibly two years, when I first saw him, April 1st, 1915. He stated that the nerve had been stretched, and massage and other treatment had been tried without benefit. Consequently he was growing despondent.

At the time of my first visit he was suffering so much pain that he could hardly rise from a sitting posture without crying out. The pain prevented sleep, and was more or less constant, becoming intensified when he used the limb. Any point along the course of the nerve was very sensitive to touch.

Ten Cc. of the one-per-cent. Solution Quinine and Urea Hydrochloride was injected into the nerve, April 1st, 1915, and for the first time in three weeks the patient slept the entire night. Injections were given on the 4th, 16th, and 29th of April. I did not see the man again until March 27th, 1916, when he informed me that he had not had any trouble since the last injection, and he had been on the road regularly all the intervening time.

Case 5.—L. M. C., 35 years old, a salesman, had a fracture of the tibia July 3rd, 1915. He would not remain in bed and went about on crutches after the fifth day, with a plaster cast on the leg. After the removal of the cast, on the twenty-third day, he began to complain of pain along the course of the sciatic nerve, which gradually grew worse.

An injection of ten Cc. of Solution Quinine and Urea Hydrochloride into the nerve on the 30th of July and a second on the 7th of August cured the malady.

Case 6.—J. D. S., a farmer, aged 34 years, was said to have had tuberculosis of the tibia when twelve years old, the diseased portion of the bone having been excised. The scar was adherent to the bone and though it would break down on very slight injury, it gave the patient no trouble.

March 3rd, 1915, when I saw him, the man was suffering from pain in the left hip-joint, but there was no rise of temperature and there were no local symptoms other than the pain. A radiogram showed a slight erosion on the head of the femur within the capsule, but no sinus was in evidence nor anything that could be interpreted as an accumulation of pus. Wasserman reaction 3 positive. The patient denied having had a specific infection, and I could find no evidenec of it beyond the condition of the hip and that afforded by the Wasserman test.

During the period from March 3rd, 1915 to February 1st, 1916, the patient received ten intravenous injections of neosalvarsan. Salvarsan or diarsenal, together with potassium iodide by mouth and mercury in intramuscular injections. A radiogram taken February first, 1916, showed that the erosion on the head of the femur had healed and the bone was in a normal condition: also the Wasserman tests were negative. The patient had ceased to feel the "catch" in the hip joint.

Since July, 1915, there had been much difficulty in holding the body erect and constant pains had developed back of the left hip joint, extending down the course of the sciatic nerve. Consequently the man walked on crutches and in a stooping attitude. In view of the findings on February first—the healing of the erosion and the negative Wasserman test—a diagnosis of sciatica was determined upon, and on March 16th, 1916, an injection of 10 Cc. of a one-per-cent Solution of Quinine and Urea Hydrochloride was made directly into the nerve. The result was that after remaining in the recumbent position for three hours he was able to stand erect for the first time in months. Whlie there was still some pain, it was not so severe and was localized below the popliteal arch. The following day, March 17th, the pain had slightly increased but the patient was able to stand erect. At this time the second injection was given. On the 18th he was free from pain, could stand straight and walk a few steps without crutches. On the 20th he had only a little pain and received the third injection. He left the city on that day with the understanding that he would return should the pain recur. A letter received from him dated March 22nd, 1916, reads: "I am still improving since leaving you. My hip, right in the joint, hurts just a little, but the leg does not bother me, and my back does not pain at. all. I know I am much better now." This patient is still under my care and will return to see me shortly. His will be an interesting case for further observation.

Since the above report, this man returned on April 1st, again complaining of a recurrence of the pain. An injection, as before, was given, and on April 3rd he reported that he was comfortable and returned to his home, to return when the pain again manifested itself. Up to the present, April 17th, he has not returned.

